# A clinical study on the relationship between chronic rhinosinusitis and bronchial asthma

**DOI:** 10.3389/fmed.2024.1388585

**Published:** 2024-10-16

**Authors:** Jing Kang, Hui Yong, Zhijuan Zhang, Jing Liu, Xiaoping Gao, Hui Shao, Li Hou

**Affiliations:** Department of Otorhinolaryngology Head and Neck Surgery, General Hospital of Ningxia Medical University, Yinchuan, China

**Keywords:** chronic rhinosinusitis, bronchial asthma, nasal polyps, inflammation, sinusitis

## Abstract

**Objective:**

To investigate the correlation between chronic rhinosinusitis (CRS) and bronchial asthma, focusing on the CRS without nasal polyps (CRSsNP) and CRS with nasal polyps (CRSwNP), as well as their impact on lung function.

**Methods:**

A total of 141 patients diagnosed with chronic nasal-sinus inflammation were included in this study. Clinical data, including medical histories, nasal endoscopy scores, CT scores, symptom scores, and quality of life assessments, were collected.

**Results:**

Among the patients with CRSsNP, 23.8% had concomitant bronchial asthma. The incidence of asthma was significantly associated with the severity of sinus involvement in CRSsNP patients (*p* = 0.049). Pulmonary function impairment was correlated with the severity of sinus inflammation in CRSsNP patients (*p* = 0.019). Quality of life was significantly affected in patients with concomitant asthma and CRSsNP or CRSwNP.

**Conclusion:**

Chronic rhinosinusitis, both with and without nasal polyps, is closely correlated with bronchial asthma. Pulmonary function impairment is associated with the extent of inflammatory lesions in CRSsNP. Although CRSwNP does not significantly affect pulmonary function, the treatment of sinus diseases can contribute to the control of asthma.

## Introduction

Chronic rhinosinusitis (CRS) refers to inflammation of the paranasal sinuses, which are air-filled spaces within the skull. According to the European Position Paper on Rhinosinusitis and Nasal Polyps (EPOS), chronic rhinosinusitis is characterized by chronic inflammation of the nasal cavity and sinuses that lasts for over 12 weeks, in this guidline, CRS is classified into primary and secondary types, further subdivided into unilateral and bilateral based on anatomical distribution (1, 2). It can be caused by viral infections such as adenovirus, parainfluenza, influenza, and rhinovirus, as well as bacterial infections including *Streptococcus pneumoniae* and *Haemophilus influenzae* (3). The prevalence rates of chronic rhinosinusitis have been reported to be as high as 11% in Europe and 12% in the United States (4, 5). In China, the prevalence of chronic rhinosinusitis ranges from approximately 2.2 to 8%, with the highest incidence observed in children aged 3–8 years (6).

Recent investigations have shed light on the pivotal role of type 2 inflammation in CRS pathogenesis. Type 2 inflammation, that’s chronic sinusitis with nasal polyps, characterized by the production of cytokines IL-4, IL-5, and IL-13, as well as the activation and recruitment of eosinophils and mast cells, plays a central role in the disease process (7, 8). Patients exhibiting a pure or mixed type 2 endotype often display resistance to conventional therapies, with higher recurrence rates compared to those with pure type 1 or 3 endotypes (9). Moreover, variations in the intensity of type 2 inflammation among patients underscore the complexity of CRS and highlight the need for tailored management and treatment approaches (10).

Based on clinical symptoms and specialized examinations for the presence of nasal polyps, chronic rhinosinusitis can be traditionallyclassified into two subtypes: chronic rhinosinusitis without nasal polyps (CRSsNP) and chronic rhinosinusitis with nasal polyps (CRSwNP) (11, 12). These two subtypes exhibit distinct clinical characteristics and immunological mechanisms of pathogenesis (13). In recent years, the relationship between nasal-sinus diseases and bronchial asthma has become a focus of research among scholars worldwide. Most researchers currently believe that there is a close association between allergic rhinitis, CRSwNP, and asthma. However, these studies mainly focus on the correlation between allergic nasal-sinus diseases and lower respiratory tract allergic diseases. Several studies have shown a higher incidence of chronic nasal-sinus inflammation in asthma patients (14–16). Chronic nasal-sinus inflammation may serve as a risk factor for asthma, impacting asthma control and disease progression (17). Wang et al. identified differentially expressed genes (DEGs) associated with the comorbidity of asthma and CRSwNP, and found an upregulation of CST1 in nasal mucosa that is related to asthma (18). In an evaluation of severe asthma patients, one study found that 45% of them had comorbid CRSwNP (19).However, there is limited research on the correlation between CRSsNP or CRSwNP and asthma, as well as lung function. This study primarily focuses on this aspect, aiming to further elucidate the relationship between them and provide more evidence for clinical treatment.

## Materials and methods

### Clinical data

A total of 141 patients diagnosed with chronic nasal-sinus inflammation and treated at the Department of Otolaryngology-Head and Neck Surgery and Respiratory Medicine of our hospital between May and December 2011 were included in this study. Among them, there were 89 males and 52 females, with an age range of 17 to 72 years (mean age: 39.2 ± 13.3 years) and an average disease duration of 8.7 ± 7.5 years. All patients provided informed consent for participation in the study.

The diagnosis of asthma is established based on the following criteria:

1. Clinical symptoms and signs of typical asthma,
  - Including recurrent episodes of wheezing, breathlessness, chest tightness, or coughing. These symptoms often occur at night and in the early morning. Patient history often reveals a correlation with exposure to allergens, cold air, physical stimuli, viral upper respiratory tract infections, and exercise.
  - During episodes, scattered or diffuse wheezing can be heard in both lungs, with a prolonged expiratory phase.
  - The aforementioned symptoms and signs can be alleviated with treatment or resolve spontaneously.
2. Objective tests indicating variable airflow limitation:
  - Positive bronchodilator test.
  - Positive bronchial provocation test.
  - An average daily peak expiratory flow (PEF) diurnal variation rate > 10% or PEF weekly variation rate > 20%.

A diagnosis of asthma is made when the above symptoms and signs are present, any of the criteria for airflow limitation are met in objective tests, and other diseases causing wheezing, breathlessness, chest tightness, and coughing are excluded.

The selection and grouping of patients were based on the following criteria:

CRSsNP group: 63 patients, including 41 males and 22 females, with an age range of 17–68 years (mean age: 35.2 ± 9.3 years) and a disease duration ranging from 3 months to 46 years (mean duration: 19.4 ± 16.3 years).

CRSwNP group: 78 patients, including 48 males and 30 females, with an age range of 22–72 years (mean age: 40.7 ± 11.3 years) and a disease duration ranging from 6 months to 50 years (mean duration: 28.6 ± 10.3 years).

### Experimental procedures

General information, including the names, genders, and ages of the selected patients, was recorded. Medical histories were collected, and specialized examinations of the ears, nose, throat, and larynx were performed to gain initial understanding of the patients’ nasal conditions. All patients underwent nasal sinus computed tomography (CT) scans, pulmonary function tests, nasal endoscopy with Lund-Kennedy scoring (20), nasal sinus CT with Lund-MacKay scoring (5, 21), visual analog scale (VAS) symptom scoring (22), and assessment of quality of life using the Medical Outcomes Study Short Form 36 (MOS SF-36) questionnaire (23).

### Preoperative symptom VAS score and nasal sinus CT score

#### VAS score

The VAS score ranged from 0 to 10, with 0 indicating no discomfort and 10 indicating severe discomfort. Patients were asked to rate their symptoms on a scale from 0 to 10 based on the severity of their symptoms, with higher scores indicating more severe symptoms. The VAS score and CT examinations were conducted within a maximum interval of 24 h.

#### LM score

The LM score evaluated nasal obstruction, with a score of 0 indicating no obstruction and a score of 2 indicating obstruction. The CT score assessed the condition of the nasal sinuses, with a score of 0 indicating normal, 1 indicating partial opacification, and 2 indicating complete opacification. The scores ranged from 0 to 12 for each side, with a total range of 0–24 for both sides. VAS scores and CT examinations were conducted within a maximum interval of 24 h.

#### Quality of life assessment

The Chinese version of the Medical Outcomes Study Short Form 36 (MOS SF-36) questionnaire, recommended by the World Health Organization (WHO), was used to assess quality of life. The questionnaire consisted of 36 items divided into eight dimensions: physical functioning (PF), role limitations due to physical health problems (RP), bodily pain (BP), general health perceptions (GH), vitality (VT), social functioning (SF), role limitations due to emotional problems (RE), and mental health (MH). Each dimension comprised 2–10 items.

### Statistics

The data were organized and analyzed using statistical methods. Descriptive statistics were presented as mean ± standard deviation (SD) for continuous variables. One-way analysis of variance (ANOVA) was used for significant difference analysis of continuous variables, while the chi-square test was used for categorical variables. A *p*-value less than 0.05 was considered statistically significant.

## Results

A total of 63 patients with CRSsNP were found to have varying degrees of bronchial asthma. Additionally, 78 patients with CRSwNP were classified into different Lund-Kennedy grades based on nasal endoscopy and nasal sinus CT examinations. The occurrence of asthma was compared between different disease ranges in the CRSsNP group using a chi-square test, showing a significant difference in the incidence of asthma (*p* = 0.049). However, there was no statistically significant difference in the incidence of asthma between different grades in the CRSwNP group (*p* = 0.943) (refer to Table 1).

**Table 1:** Incidence of asthma in both groups *n* (%).

	*n*	Bronchial asthman (%)	*p*
CRSsNP			0.049
Single sinusitis	7	0 (0)	
Multiple sinusitis	44	8 (18.2)	
Pansinusitis	12	7 (58.3)	
Total	63	15 (23.8)	
CRSwNP			0.943
(Lund-Kennedy)I	7	2 (28.5)	
II	18	6 (33.3)	
III	31	7 (27.3)	
IV	22	6 (26.9)	
Total	78	21 (26.9)	

CRSsNP, chronic rhinosinusitis without nasal polyps; CRSwNP, chronic rhinosinusitis with nasal polyps.

The comparison of asthma incidence between the CRSsNP and CRSwNP groups was analyzed using a chi-square test, revealing no statistically significant difference in the incidence of asthma between the two groups (*p* = 0.745) (see Table 2).

**Table 2:** Comparison of prevalence of asthma in CRSsNP and CRSwNP *n* (%).

	*n*	Bronchial asthman (%)
CRSsNP	63	15 (23.8)
CRSwNP	78	21 (26.9)
*p*		0.745

CRSsNP, chronic rhinosinusitis without nasal polyps; CRSwNP, chronic rhinosinusitis with nasal polyps.

Among the 15 patients with CRSsNP and concomitant bronchial asthma, a comparison of different disease ranges in the nasal sinuses and the degree of pulmonary function impairment showed a statistically significant difference (*p* = 0.019). However, among the 21 patients with CRSwNP and concomitant bronchial asthma, there was no statistically significant difference in pulmonary function changes between different grades of nasal polyps (*p* = 0.214) (see Table 3).

**Table 3:** Incidence of pulmonary dysfunction with asthma in both groups *n* (%).

	*n*	Small airway obstructive ventilatory dysfunction (%)	Pulmonary ventilation obstructive dysfunction (%)	*p*
CRSsNP				0.019
Single sinusitis	0	0 (0)	0	
Multiple sinusitis	8	6 (75)	2 (25)	
Pansinusitis	7	1 (14.2)	6 (85.8)	
Total	15	7 (46.7)	8 (53.3)	
CRSwNP				0.214
(Lund-Kennedy) I	2	2 (100)	0	
II	6	5 (83.3)	1 (16.7)	
III	7	4 (57.1)	3 (42.9)	
IV	6	2 (33.3)	4 (66.7)	
Total	21	13 (61.9)	8 (38.1)	

The comparison of pulmonary function impairment between patients with concomitant bronchial asthma in the CRSsNP and CRSwNP groups showed no statistically significant difference (*p* = 0.364) (see Table 4).

**Table 4:** Comparison of the incidence of pulmonary dysfunction with asthma between the two groups of patients.

	*n*	Small airway obstructive ventilatory dysfunction (%)	Pulmonary ventilation obstructive dysfunction (%)
CRSsNP	15	7 (46.7)	8 (53.3)
CRSwNP	21	13 (61.9)	8 (38.1)
*p*		0.364	

CRSsNP, chronic rhinosinusitis without nasal polyps; CRSwNP, chronic rhinosinusitis with nasal polyps.

In this study, independent sample t-tests were used to compare certain clinical indicators in patients with concomitant bronchial asthma. It was found that there was no statistically significant difference in nasal endoscopic findings and imaging examinations. However, patients’ subjective symptoms and impact on quality of life showed significant differences (see Table 5).

**Table 5:** Analysis of clinical indicators.

	CRSsNP	CRSsNP + Asthma	*p*	CRSwNP	CRSwNP + Asthma	p*
Nasal endoscopy score	4.78 ± 1.32	4.93 ± 1.74	0.71	8.42 ± 1.39	8.93 ± 1.28	0.13
Sinus CT score	14.98 ± 1.15	15.12 ± 1.32	0.68	16.21 ± 1.53	16.33 ± 1.17	0.74
Symptom score	6.00 ± 1.22	7.79 ± 1.02	<0.001	6.62 ± 1.46	8.13 ± 1.94	0.002
SF nasal-sinusitis 36 score						
PF	78.57 ± 14.11	79.35 ± 15.63	0.001	65.37 ± 12.38	66.72 ± 13.72	0.007
RP	88.68 ± 31.15	71.23 ± 9.53	0.01	82.36 ± 28.37	69.33 ± 17.47	0.026
BP	54.08 ± 18.23	49.21 ± 12.35	<0.001	35.38 ± 15.63	31.33 ± 11.43	<0.001
GH	86.27 ± 11.43	85.14 ± 11.23	<0.001	70.20 ± 14.43	71.08 ± 13.17	<0.001
VT	58.30 ± 8.79	61.23 ± 7.53	<0.001	43.26 ± 7.27	45.63 ± 6.29	<0.001
SF	46.78 ± 17.15	49.57 ± 16.86	0.006	33.21 ± 14.32	31.48 ± 16.23	0.001
RE	66.09 ± 16.27	60.21 ± 14.34	0.001	50.54 ± 10.44	45.21 ± 13.58	0.001
MH	87.16 ± 14.87	86.67 ± 13.76	<0.001	60.74 ± 16.31	57.55 ± 12.84	<0.001

PF, Physical Functioning; RP, Role-Physical; BP, Bodily Pain; GH, General Health; VT, Vitality; SF, Social Functioning; RE, Role-Emotional; MH, Mental Health. *p*, Comparison of CRSsNP with CRSsNP+ Asthma. p*, Comparison of CRSwNP with CRSwNP + Asthma. CRSsNP, chronic rhinosinusitis without nasal polyps; CRSwNP, chronic rhinosinusitis with nasal polyps.

## Discussion

The upper and lower respiratory tracts are interconnected and functionally related. Clinically, allergic rhinitis often coexists with sinusitis and nasal polyps, affecting lower respiratory tract diseases (24, 25). Bronchial asthma is characterized by chronic allergic inflammation of the airways, involving multiple genes and exhibiting genetic susceptibility (26, 27). In this study, among 63 patients with chronic rhinosinusitis without nasal polyps (CRSsNP), 15 patients were found to have concomitant asthma. Comparative analysis revealed a correlation between the incidence of asthma and the severity of chronic rhinosinusitis without nasal polyps. The wider the range of sinus involvement, the higher the incidence of asthma. It is postulated that when patients have more severe sinus inflammation, there is an increased quantity of bacterial superantigens produced by microbial toxins, leading to a more pronounced airway inflammatory response. This indicates the presence of lower airway inflammation and systemic inflammation (28, 29). The results of this study further support the close association between sinusitis and asthma. The severity of sinusitis influences the occurrence and development of asthma. Patients with concomitant chronic rhinosinusitis without nasal polyps and asthma are more susceptible to viral infections (30). This suggests that sinus inflammation may make asthma patients more vulnerable to viral invasion, leading to recurrent asthma symptoms that are difficult to control.

By analyzing the data of 15 patients with chronic rhinosinusitis without nasal polyps and concomitant bronchial asthma, this study demonstrated that as the severity of sinus inflammation increased, pulmonary function deteriorated. This was manifested by a significant increase in airway resistance, a decrease in forced vital capacity, forced expiratory volume in 1 s, and peak expiratory flow rate, and an increase in residual volume. These findings suggest a close correlation between the extent of inflammatory lesions in chronic rhinosinusitis without nasal polyps and the severity of bronchial asthma. Some researchers have evaluated the medical history, histamine provocation test, and pulmonary function and concluded that 60% of patients with chronic rhinosinusitis without nasal polyps have lower respiratory tract involvement (31). In our study, the incidence of asthma among patients with chronic rhinosinusitis without nasal polyps was 23.8%, which differs from the findings of some previous studies. This discrepancy may be attributed to the relatively small sample size in our study and the fact that we focused on the relationship between sinusitis and bronchial asthma, without considering other lower respiratory tract inflammations such as bronchitis.

The pathogenesis of nasal polyps remains unclear. However, studies have shown that patients with nasal polyps have increased airway responsiveness, and chronic rhinosinusitis with nasal polyps (CRSwNP) is considered the most closely related nasal sinus disease to asthma from an epidemiological, clinical, and pathological perspective (32). The results of our study showed that the incidence of bronchial asthma in the CRSwNP group was 26.9%, and there was no significant difference in the incidence of asthma among patients with different grades of nasal polyps. From Table 3, it can be observed that there was no significant statistical difference in the degree of pulmonary function changes among patients with nasal polyps of different grades, suggesting that the severity of disease in patients with chronic rhinosinusitis with nasal polyps is not significantly correlated with the severity of asthma. However, the treatment of sinus diseases can contribute to the control of asthma. Multiple studies have shown that medication treatment (such as omalizumab and benralizumab) or nasal polyp removal can improve the quality of life in asthma patients (33, 34). Additionally, allergen immunotherapy (AIT) can modify the immune response and reduce inflammation (35). These treatment modalities address the underlying inflammatory processes, offering a comprehensive approach to managing patients with comorbid chronic rhinosinusitis and asthma. Further research into these treatments and their impact on both conditions is essential for optimizing patient outcomes.

The results of this study indicate that there was no statistically significant difference in the incidence of bronchial asthma between the CRSwNP group and CRSsNP group. This suggests that although the pathogenesis of CRSsNP and CRSwNP may differ, they have a common impact on bronchial asthma. This further supports the notion of interconnection between the upper and lower respiratory tracts and the shared effects of different upper respiratory inflammatory diseases on the lower airways. The findings of this study also demonstrate a decline in overall quality of life in patients with concurrent asthma and CRSsNP or CRSwNP, particularly in terms of physical functioning, bodily discomfort, and mental health. Similar results have been reported in several other studies (36–38).

One limitation of this study is the relatively small sample size, which may affect the generalizability of the findings. Additionally, the study focused on the relationship between chronic rhinosinusitis (CRS) and bronchial asthma, without considering other potential factors that could influence the development and severity of asthma, such as genetic predisposition or environmental factors. Furthermore, the study did not explore the long-term outcomes or the effects of different treatment interventions on the management of CRS and asthma. Future research with larger sample sizes and a more comprehensive approach is needed to further investigate the relationship between CRS and asthma and to provide more robust evidence for clinical treatment.

## Data Availability

The original contributions presented in the study are included in the article/supplementary material, further inquiries can be directed to the corresponding author.
